# Stochastic lithofacies and petrophysical property modeling for fast history matching in heterogeneous clastic reservoir applications

**DOI:** 10.1038/s41598-023-50853-3

**Published:** 2024-01-02

**Authors:** Watheq J. Al-Mudhafar, Hung Vo Thanh, David A. Wood, Baehyun Min

**Affiliations:** 1 Basrah Oil Company, Basrah, Iraq; 2 Laboratory for Computational Mechanics, Institute for Computational Science and Artificial Intelligence, Van Lang University, Ho Chi Minh City, Vietnam; 3 Faculty of Mechanical-Electrical and Computer Engineering, School of Technology, Van Lang University, Ho Chi Minh City, Vietnam; 4 DWA Energy Limited, Lincoln, UK; 5 Center for Climate/Environment Change Prediction Research, Ewha Womans University, 52, Ewhayeodae-gil, Seodaemun-gu, Seoul, 03760 Republic of Korea; 6 Department of Climate and Energy Systems Engineering, Ewha Womans University, 52, Ewhayeodae-gil, Seodaemun-gu, Seoul, 03760 Republic of Korea

**Keywords:** Energy infrastructure, Fossil fuels, Hydrology

## Abstract

For complex and multi-layered clastic oil reservoir formations, modeling lithofacies and petrophysical parameters is essential for reservoir characterization, history matching, and uncertainty quantification. This study introduces a real oilfield case study that conducted high-resolution geostatistical modeling of 3D lithofacies and petrophysical properties for rapid and reliable history matching of the Luhais oil reservoir in southern Iraq. For capturing the reservoir's tidal depositional setting using data collected from 47 wells, the lithofacies distribution (sand, shaly sand, and shale) of a 3D geomodel was constructed using sequential indicator simulation (SISIM). Based on the lithofacies modeling results, 50 sets of porosity and permeability distributions were generated using sequential Gaussian simulation (SGSIM) to provide insight into the spatial geological uncertainty and stochastic history matching. For each rock type, distinct variograms were created in the 0° azimuth direction, representing the shoreface line. The standard deviation between every pair of spatial realizations justified the number of variograms employed. An upscaled version of the geomodel, incorporating the lithofacies, permeability, and porosity, was used to construct a reservoir-flow model capable of providing rapid, accurate, and reliable production history matching, including well and field production rates.

## Introduction

The primary goal of oil/gas reservoir modeling is to reliably forecast future field production and resource-recovery performance to assist with field development decisions. Field development typically involves the consideration of several alternative scenarios. A reliable simulator makes it possible to test and select the optimum scenario to achieve various objectives, such as infill drilling (i.e., the number of wells to be drilled, when, and at which location), waterflooding (i.e., the timing and number of injection wells at which location), and other production management options. This information helps to maximize oil/gas recovery from the simulated reservoirs^1^. Dynamic reservoir simulation typically solves sets of differential equations related to the established reservoir properties using a 3D grid model to simulate fluid flow over time^2^. In particular, a full-physics reservoir simulation is required to analyze the reservoir and its dynamic fluid flow and thus accurately forecast and plan future reservoir performance. With this goal in mind, this study aimed to conduct detailed reservoir lithofacies and flow-modeling analysis to evaluate the fluid flow through the upper clastic member of the Zubair formation (Lower Cretaceous) for the Luhais onshore oil field located in southern Iraq.

A key challenge in reservoir modeling is the integration of static and dynamic data. Typically, static data is spatially limited across reservoirs and is derived from various sources at different scales. Consequently, it is often worthwhile to employ geostatistical quantification to compile this data and reduce uncertainty in the value distributions. The lithology also influences the reservoir fluid characteristics based on differences in the capillary pressure and relative permeability profiles. These profiles impact the decision-making regarding the productivity and the financial feasibility of reservoir and field development^3^.

Effective facies modeling requires that the available petrophysical data and facies types are combined with the structural architecture of the reservoir in a 3D representation. This ultimately needs to involve all flow units present in a dynamic simulation. Multi-scale data also needs to be included to capture the degree of heterogeneity associated with the reservoir zones of interest. Deterministic information can be complemented by stochastic simulation techniques to better quantify the facies and petrophysical parameters for a reservoir using data-driven methods^4^.

In this context, pixel-based image datasets consisting of both discrete and continuous variables are now widely used geostatistical frameworks that become input for stochastic models. Sequential indicator simulation (SISIM) is a pixel-based technique suitable for facies modeling, while sequential Gaussian simulation (SGSIM) is widely employed for petrophysical properties. Other approaches can be used for modeling facies, including object-oriented and multi-point simulators^5,6^. Pixelated images are generated pixel-by-pixel while accounting for nonparametric conditional probabilities. Variograms compile the variance between pairs of spatially distributed points across the reservoir zone^7^, applying what is referred to as a two-point statistical technique^8^. However, although variogram-based modeling is easy to apply, it requires only a few parameters and is thus limited for certain simulation applications. For example, it struggles to sufficiently capture certain complex geological features, including channel forms, thickness variations, and sinuosity. Indeed, several geological features remain undetected within a single variogram^9^.

SISIM has been successfully employed across a range of reservoir lithologies, particularly sandstones, to characterize various spatial patterns, including sinuous and planar features (e.g., flow channels and faults)^10^, complicated deep-water turbiditic sequences^11^, and tidal depositional environments^12^. SISIM models can combine multi-scale data from multiple sources, including well logs, geophysical surveys, and lithofacies analysis^13^. SISIM generates a conditional probabilistic model that captures the stochastic characteristics of lithologies via ternary modeling^14^. In addition, many scholars^15–18^ have applied the Sequential Indicator Simulation (SIS) technique, incorporating alterations pertaining to different estimation methods and the incorporation of secondary variables.

Categorical variables can be readily processed as a sequence of numbers representing each class and then modeled as variograms or covariance functions and displayed spatially in map form. Lithofacies data can be represented in SISIM as elemental segments (0*–*1), which are regulated by set standard limits (i.e., thresholds). The spatial patterns of these observations are described in the form of variograms, which are used to generate stochastic maps, contoured in terms of probability, of the reservoir zones.

Binary code can be used to distinguish between different lithology types, such as shale and sand^19^. These lithofacies can be represented as a set of variables in space by allocating numbers to specific locations, such as 1 for a sand channel and 0 for a turbidite sandstone lobe^20^. By interpolating the electro-facies of available well logs, SISIM can identify lithofacies variation between wells. The electro-facies information also enables SISIM to incorporate uncored boreholes in the lithofacies distribution based on conditional stochastic interpretation. SISIM can be adjusted vertically using the mean contribution of specific lithofacies to stochastically identify transitions from marine to non-marine depositional environments^21,22^.

SISIM has been exploited for a wide range of reservoir characterization purposes^23,24^. In this context, porosity and permeability properties need to be spatially established because they are the primary factors influencing the relative flow of fluids through reservoir zones. Here, variograms can be exploited to spatially define porosity–permeability distributions, thus providing insights into reservoir geometry^25^. Kriging techniques^26^ are also useful for this purpose because they can assist in determining best-linear-unbiased estimates and/or predictions (BLUE/BLUP)^27^. In particular, kriging combined with SGSIM or its truncated modification provides useful spatial computations^28^ of petrophysical parameters when constructing 3D geomodels incorporating stochastically derived porosity–permeability information^29^.

Variograms provide useful information on reservoir geometry, particularly when combined with kriging to ensure spatial precision^29^. Kriging quantifies the relationship between predictable and unpredictable data in order to predict missing values based on available data at neighboring locations^30^. Co-kriging makes it possible to estimate two factors simultaneously, for example utilizing porosity–permeability correlations, predicting one variable as a function of another^31^. In addition, if a measured distribution of a secondary petrophysical parameter or seismic attribute is available, a variable of interest (e.g., porosity) can be approximated as a principle component in some circumstances using collocated co-kriging^32^. However, instead of depending on a single assessment using kriging extrapolation, stochastic modeling can be effective in generating numerous geological realizations of many petrophysical properties^33^. SGSIM is the most commonly used approach to determine the properties of continuous distributions. Spatial information can be translated into a normally distributed variable, based on which conditional modeling computes and fits variograms^34^. SGSIM then estimates the properties by applying conditional probabilities to each grid point and randomly sampling the constructed normally distributed variable^35,36^.

As an inverse problem, history matching has deployed various techniques that are sometimes deterministic or stochastic, minimization or sampling, considering given circumstances^37^. A recent study employed an integrated modeling framework to improve the accuracy of history-matched models in fluvial channel reservoirs and introduced a modeling workflow capable of achieving rapid history matching for dynamic simulation models^38^. Integrated history matching has also been proposed to improve production forecasting for an unconventional gas field in the Surat basin, Australia^39^. In that study, the uncertainty associated with multiple parameters was considered before conducting the history-matching process, in which only the most influential parameters for history matching and field production forecasting were selected. Almeida et al.^40^ also demonstrated that reservoir uncertainties strongly impacted both history matching and production forecasting results for a planned oil field development. In addition, integrated modeling procedures have been conducted to assist history matching in fractured reservoirs, with a history-matched model used to optimize well spacing for shale gas formations in China by including uncertainty in geological variables and economic feasibility^41^. In other recent studies, geological facies models have been constructed using self-attention generative adversarial networks^42,43^ but these studies developed models based on synthetic reservoirs. A significant challenge for the simulation modeling of complex reservoirs is to effectively apply the resulting models to real field datasets. Thus, the efficiency of the simulation modeling process for gas and oil exploration and exploitation needs to be taken into account.

Although geostatistical modeling has been well established, it has rarely been applied to large oil/gas field reservoir modeling, particularly in applying the geostatistical techniques to a real field case study described on an integrated basis. For this reason, the present study employed a multi-faceted geostatistical technique to Luhais, one of the largest onshore oil field in southern Iraq. The key objectives of this study were as follows:

- An efficient geostatistical modeling framework of integrated facies and petrophysical properties was developed to allow for rapid reservoir history matching for use in field development planning and decision-making.
- The uncertainty associated with the estimates of static reservoir properties was reduced by employing a more integrated geostatistical modeling framework.

This study is thus novel in that it proposed systematic geostatistical modeling for rapid history matching based on the production and injection profile for the entire history. Geological uncertainty was also taken into account to improve the history-matching capabilities of reservoir simulation models.

## Methodology

**Figure 1:**
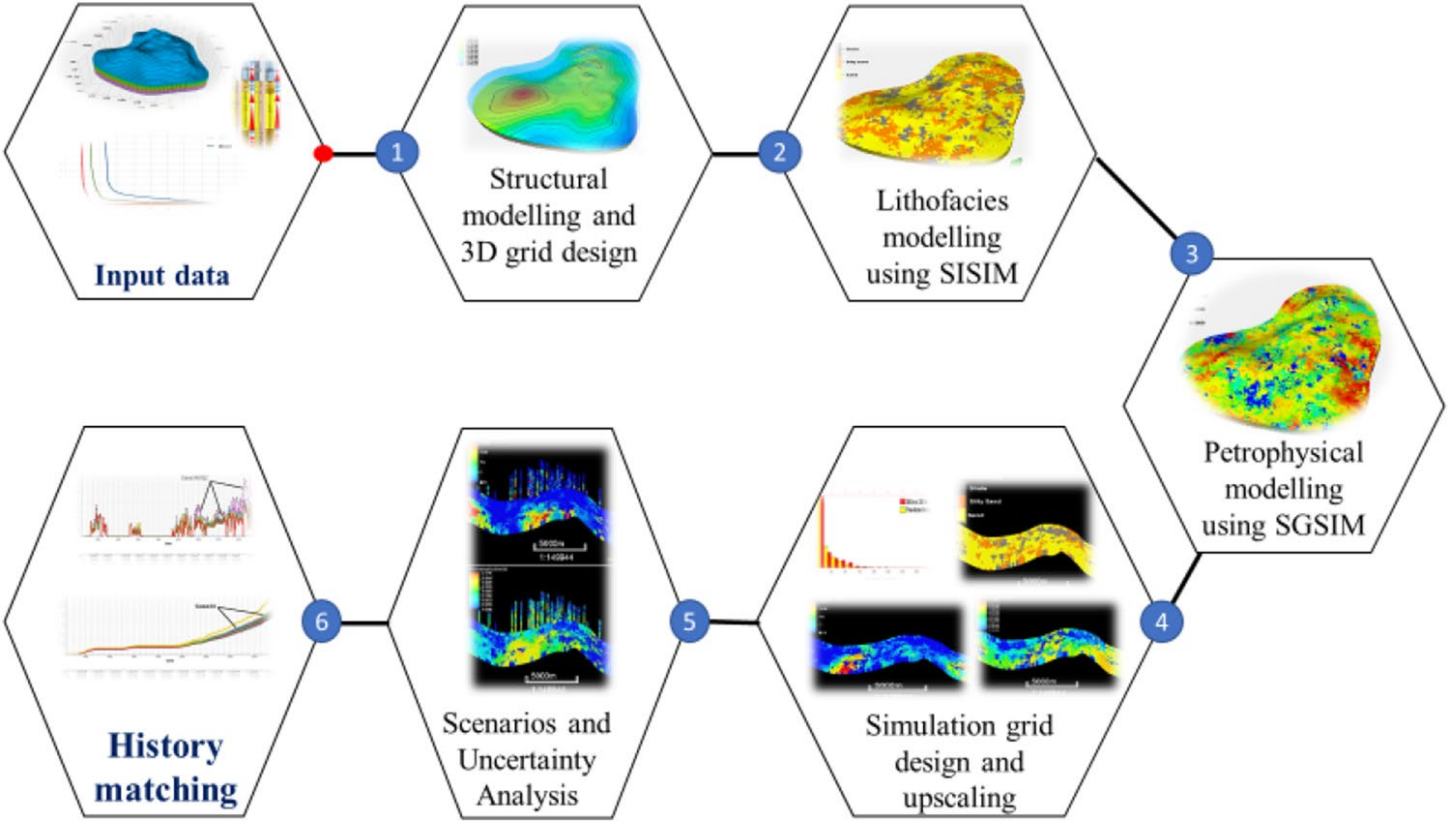
Workflow used to develop the integrated geostatistical model for improved oil and gas reservoir simulation.

Figure 1 presents the workflow associated with the integrated geostatistical modeling framework developed in the present study. Initially, the geological data available for the Luhais oilfield was compiled, including core data collected from 1 well and well-log data from 47 wells that penetrate the target reservoir formation. SISIM was then used to establish a lithofacies model to derive information on the reservoir-wide lithology and facies distribution. SGSIM was then employed for petrophysical analysis to delineate the geological heterogeneity and anisotropy across the reservoir. Subsequently, a multi-phase numerical reservoir simulation model was applied to all of the constructed static geological models to identify the main parameters influencing the reservoir fluid flow. This allowed the development of a history-matching reservoir simulation model based on porosity–permeability distributions, rock compressibility, aquifer properties, capillary pressure, and relative permeability curves as the key parameters. The developed integrated models also considered spatial heterogeneity/reservoir anisotropy and uncertainty analysis.

### Stochastic lithofacies modelling

For a spatial lithofacies model to be useful, all of the lithological components need to be represented, thus the 3D geometry of the sedimentary subsurface architecture must be correctly depicted. Lithofacies information can also indicate the various depositional settings that are present and represent the overall spatial heterogeneity for eventual coupling with hydrodynamic models of fluid flow^6,44^. By managing the subsurface inhomogeneity and hydraulic flow parameters, lithofacies models can be used to simulate reservoir features at specific spatial and depth locations within a reservoir unit^45^.

In SISIM, each node is allocated randomized lithotype *k* from a discrete distribution based on the indicator variogram for each lithofacies, which are encoded with values of 0 or 1 based on assigned threshold values (Eq. 1)^23^:

1 $$I ({Z}_{k};x)=\left\{\begin{array}{cc}0& if Z\left(x\right)>{Z}_{k} \\ 1& if Z\left(x\right)\le {Z}_{k}\end{array}\right\}$$

where $I ({Z}_{k};x)$ is a random indicator*, Z (x*) is a random function, and ${Z}_{k}$ is the assigned threshold value. $I ({Z}_{k};x)$ has an expected value equal to the accumulated likelihood *Pr* $\left\{Z\left(x\right)<{Z}_{k}\right\}$ of its random indicator as defined in Eqs. (2 and 3):

2 $$E\left(I\left({Z}_{k};x\right)\right)=0\times Pr\left\{Z\left(x\right)>{Z}_{k}\right\}+1\times Pr\left\{Z\left(x\right)\le {Z}_{k}\right\}$$

3 $$E\left(I\left({Z}_{k};x\right)\right)= Pr\left\{Z\left(x\right)<{Z}_{k}\right\}$$

SISM utilizes Eq. (3) for non-parametric sequential simulations. Once a grid skeleton and coordinate system are established, the SISM model can be derived as follows^46^:

- Lithofacies data (*K*) is converted into binary coded indicators (*I*) (Eq. 3)
- An indicative variogram is generated based on the binary code for every lag interval as in Eq. (4):
  4 $$\gamma (h)=\frac{1}{2{N}_{h}}\sum_{i=1}^{{N}_{n}}\left({facies}_{(h+1)}-{facies}_{h}\right)$$
  where $\gamma (h)$ is the variogram as a function of lag distance *h*, and *N*_*h*_ is the number of points included in the indicator variogram. The lag interval (or lag distance) *h* represents the distance at which the spatial points are correlated.

The density function, which describes the dispersion of the facies, is calculated using Eq. (5):

5 $$F({z}_{i})=\sum_{j=1}^{i-1}P({z}_{j})$$

where $F({z}_{i})$ represents the prior distribution function of the facies, and $P({z}_{j})$ is the density distribution of the facies.

- Extrapolation is conducted by evaluating all of the unsampled areas at random.
- Kriging of the indicator values is conducted to predict a predominate lithofacies at an unsampled area considering the status of the indicator in the surrounding areas.
- The indicator is set at 0 or 1 at random.
- This process is continued for the remainder of the unsampled locations after incorporating the modeled indicator values for the sampled dataset.
- The process is repeated to generate multiple stochastic reservoir images (i.e., realizations).

### Stochastic petrophysical property modelling

SGSIM is preferred to truncated Gaussian simulation for porosity–permeability modeling because it uses an accumulated probabilistic model to recognize only the data inside a defined search region. Once a variogram is constructed, SGSIM applies a basic kriging algorithm to establish the initial data points. The variogram assists in the geometric understanding of the continuity and heterogeneity of reservoir parameters, which have a direct influence on fluid-flow behavior. Unlike simple kriging, the SGSIM stochastically generates numerous equiprobable stochastic reservoir models (i.e., realizations) by randomly selecting unsampled locations driven initially by a seed number. The estimated values at the randomly selected locations are utilized to predict values at other unsampled reservoir locations. The major steps involved in conditional SGSIM are as follows^47^:

- Sampled data variables are transformed into Gaussian distributions (mean µ = 0; variance σ^2^ = 1) via Z-score transformation.
- Variograms are constructed.
- Reservoir locations are randomly selected using a seed number.
- The values of the variables at the selected locations are determined and the associated error is estimated based on the variance derived from kriging.
- Local cumulative probability functions are established for each estimated variable in each local area.
- The previous steps are applied to the remaining locations for a specific realization.
- All of these steps are repeated to generate multiple realizations.

### Dynamic reservoir simulation process

The dynamic simulation employed in this study adopts the steps proposed by Aziz and Settari^1^:

- The specific reservoir challenges and their commercial implications are determined.
- The relevant static reservoir properties and dynamic influences on fluid flow are determined.
- Geological, geophysical, and petrophysical data and their interpretation are combined to develop 3D geological models.
- Pressure–volume–temperature (PVT) analysis of reservoir fluid samples is conducted.
- Simulator software is selected.
- The reservoir grid is configured and scaled and the time step intervals to be modeled are selected.
- Sensitivity analysis is conducted to establish the values for key simulator control variables.
- The simulator is configured for reservoir production history-matching analysis.
- The dynamic simulation is employed to forecast reservoir production profiles with the use of different infill drilling programs conducted at different future dates in order to establish the optimal reservoir development plan.

These steps aim to produce the optimal history-matching model for the complex tidal clastic reservoir in the Luhais oil field in southern Iraq.

### Reservoir production history matching process

A key output of credible reservoir-flow simulations is history matching because it not only verifies the efficacy of the model but can also be used to forecast future production profiles. Maintaining the consistency between observed and predicted fluid flows from a reservoir simulation is an important inversion step, allowing the fine-tuning of the reservoir model’s assumptions by adjusting them to optimize the match between historically recorded and predicted production profiles. It is not only production rates that should be matched; reservoir pressure and fluid saturation trends must also be considered. Once accurate history matches are established for these variables, the simulation model can be used to generate predictions for forward-looking reservoir trends, thus assisting with reservoir development decisions. Accurate history matching is a difficult process, particularly for complex heterogeneous reservoirs. It typically takes multiple trial-and-error runs of a simulator, often with the aid of an optimizer, to reduce the history matching errors to acceptable levels. The unknown levels of uncertainty associated with spatially extrapolated reservoir variables exacerbate the difficulties of reservoir history matching.

Simulation mismatch errors are often determined in terms of a weighted-sum-of-squares difference between measured values ***Y***^*obs*^ and simulated predicted/calculated values ***Y***^*cal*^ (Eq. 6)^9^:

6 $$Q(x)=\sum_{i=1}^{{n}_{obs}}{{w}_{i}\left({Y}^{obs}-{Y}^{cal}\right)}^{2}$$

where $Q(x)$ is the mismatch error (observed versus simulated) for history-match variable *x*, ${w}_{i}$ is the mismatch weight factor, ${Y}^{obs}$ is the distribution of observed values for variable *x* (*n* observations), and ${Y}^{cal}$ is the distribution of simulated values for variable *x* (*n* observations).

Equation (6) was used to assess the simulation mismatch errors in the present study, while the entire workflow of geological modeling and reservoir flow simulation was implemented using the Petrel and Eclipse packages^48^.

## Field description

### Geographical description

**Figure 2:**
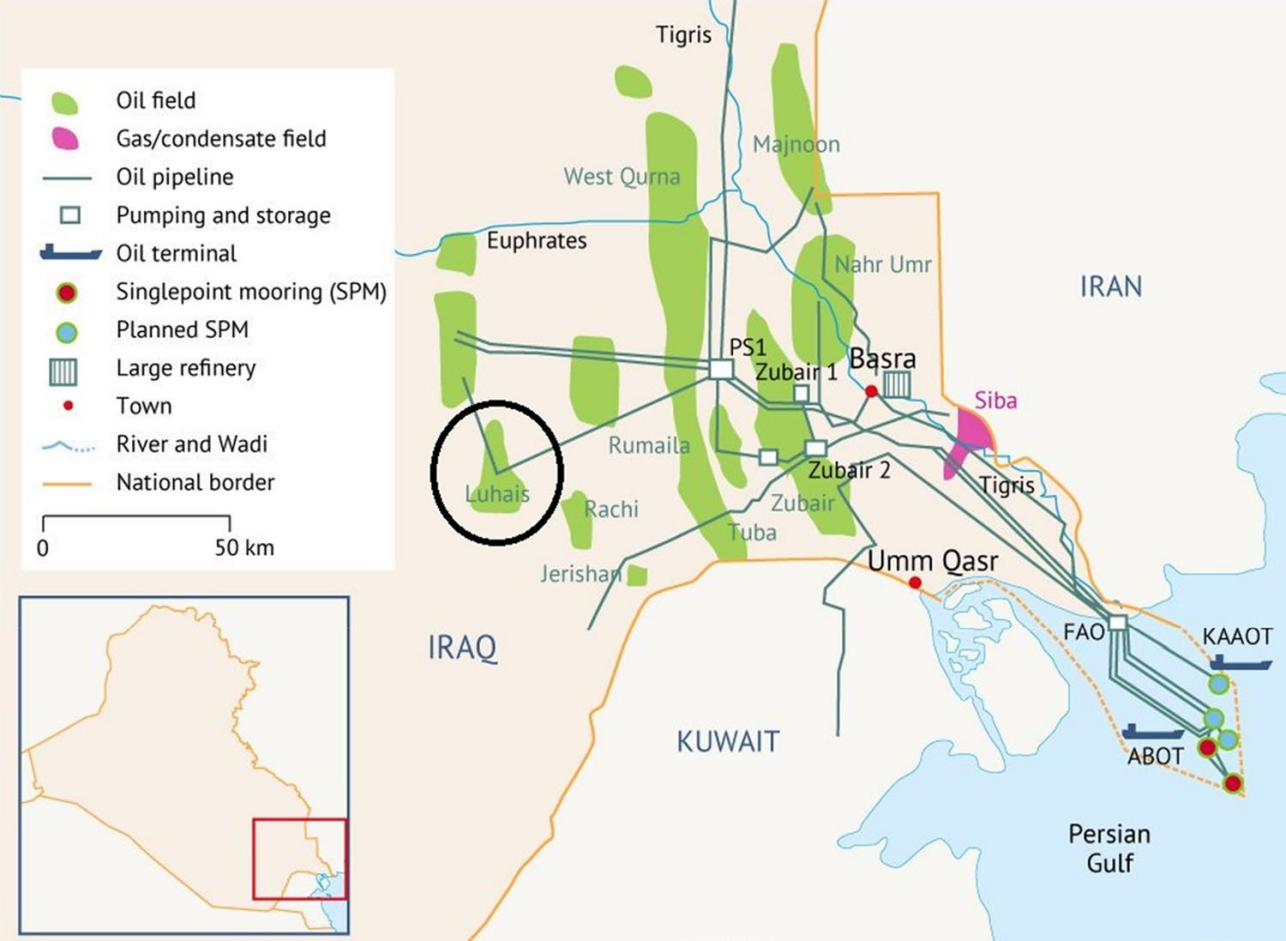
Location and features of the Luhais oil field, Iraq^50^.

The Luhais oil field is one of the most productive fields in southern Iraq (Fig. 2). This large field, which was initially appraised by BP in 1961^49^, is located 105 km northwest of Basra and 80 km north of the Rumaila oil field. Luhais is part of a suite of large oil fields extending to the north and east (Fig. 2). The field is 20 km long (north to south) and 5 km wide in the north, broadening to a width of about 10 km in its central and southern sections^50^. It consists of a gently dipping anticline with a northwest–southeast trending axis, with gentle slopes dipping 1° on its western flank and 2*.*5° on its eastern flank.

### Geological background

The Luhais oil field produces hydrocarbons from the Zubair and Nahr Umar geological formations^49^. Glynn Jones assigned the name “Zubair” to a sandstone formation in the Zubair oil field in 1948^51^. This Lower Cretaceous formation has become one of the most prolific classic reservoirs in the region^52^, particularly in southern Iraq^53^. The Zubair’s clastic sequence consists mainly of sandstones deposited in fluvial-deltaic and marine environments^49^. Reservoir contour maps of the upper member of the Zubair formation in the Luhais field reveal an asymmetric anticlinal fold with several domal culminations with no faulting^50^.

**Figure 3:**
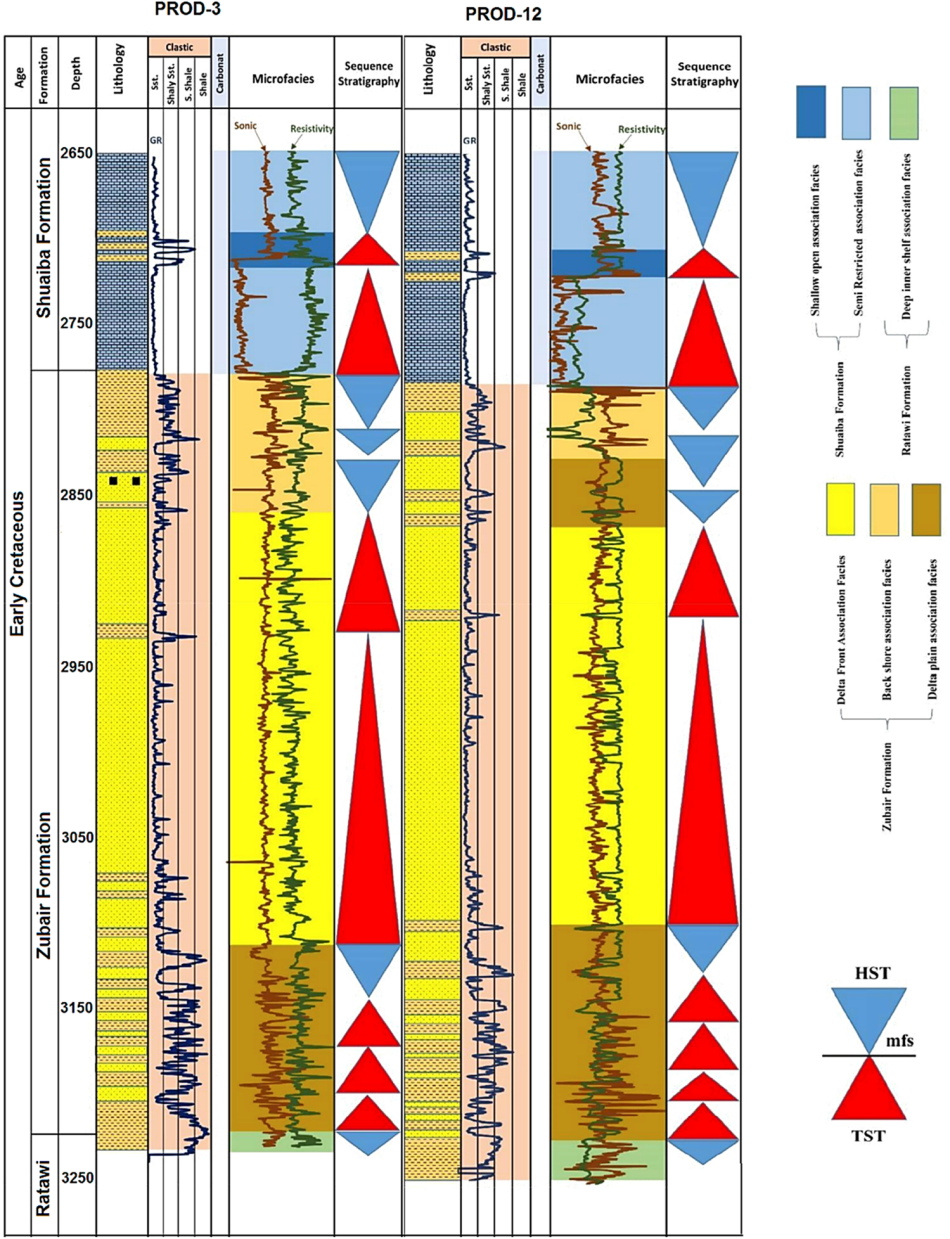
Stratigraphic details of a geologic section of the Luhais oil field/Mesopotamian basin^54^.

Figure 3 illustrates the stratigraphic column of the Luhais oil field^54^. Across the Luhais field, the Zubair formation varies in depth from 2777 to 3227 m, with an average thickness of about 450 m^49^. Based on petrophysical data collected from wells, the Zubair formation has been categorized into five distinctive groups: (i) upper shale, (ii) upper sandstone, (iii) middle shale, (iv) lower sandstone, and (v) lower shale. The upper shale group of the Zubair is mainly composed of sandstone, shale, and siltstone strata, with thin carbonate layers. The vertical lithology distribution in the upper shale group consists of a thick sandstone member sandwiched between thinner upper and lower shale members. The upper shale member is the main oil pay of the Luhais field because it consists of pure sand in most wells, which is interbedded with some shale and silt layers. This member has been deposited in a regressive tidal depositional environment, where multiple fluvial streams have been formed, each with limited spatial distribution^54^. There are some thin sandstone and siltstone interbeds in the upper and lower shale members.

### Static and dynamic reservoir data for the Zubair formation in the Luhais field

Dynamic data available from the Luhais field that was analyzed in the present study included geological and petrophysical data from the wellbore, the historical dates of when each well was perforated at specific depth intervals in the reservoir zones, the completion status and production history of each well, and PVT data for the formation fluids recovered downhole during well tests. The PVT data provided details on the initial solution gas-to-oil ratio (GOR) in the reservoir and how it evolved during the oil field’s production history. PVT data was used to establish the formation volume factor and fluid viscosity, which are required for accurate black oil simulation analysis, and to generate capillary-pressure relationships across the reservoir, while relative-permeability curves for each lithofacies were also established.

The physical and thermal parameters of reservoir fluids acquired from the Luhais oil field reservoirs were calculated using data derived from a substantial number of fluid samples collected over the field’s production life. The upper shale member of the Luhais oil field is an undersaturated water–oil reservoir whose initial and current reservoir pressures are above the bubble point pressure of 2300 psia. The initial reservoir pressure is 4300 psia, recorded at a depth of 2735 m. The initial oil–water contact (OWC) is at a TVD of 2755 m. Table 1 summarizes the reservoir properties taken from three wells in the Luhais oil field/upper shale member.

**Table 1:** Upper shale oil reservoir properties of the Luhais field taken from the testing of three wells.

Well	Sampling date	API	Perf (m)	BHP (psia)	Pb (psia)	T (°F)	GOR (SCF/STB)	Bo (bbl/STB)
PROD-06	30/5/2011	31.8	2809	4279	2411	185	630.5	1.3932
PROD-17	14/3/2011	32.4	2796	4233	2264	185	620.0	1.4035
PROD-36	14/10/2011	32.1	2803.5	4257	2293	83.2	610.6	1.3968

**Figure 4:**
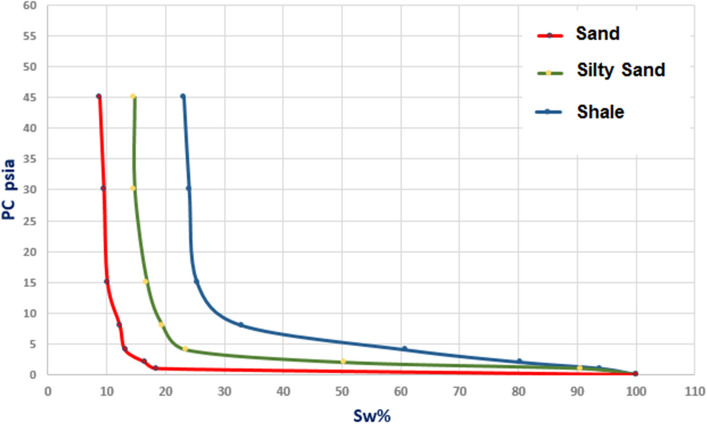
Capillary pressure curves for the upper shale member in the Luhais oil field.

**Figure 5:**
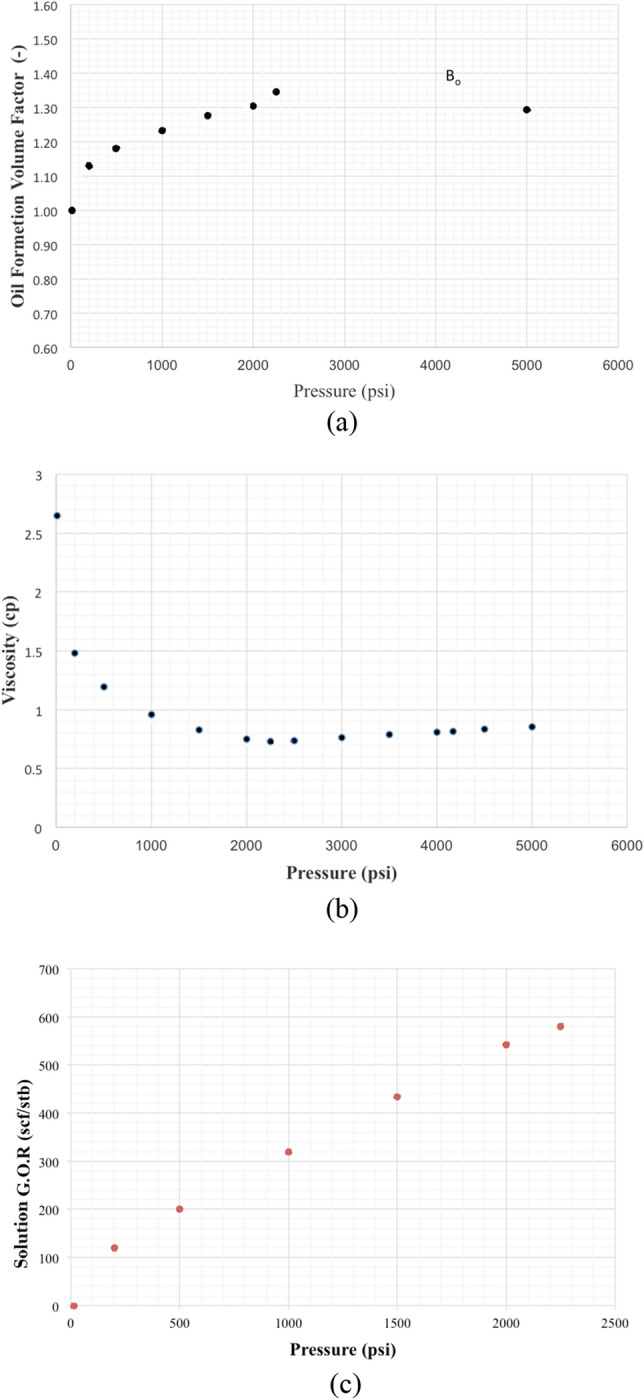
PVT data used for the reservoir simulation of the Luhais oil field model: (**a**) oil formation volume factor, (**b**) oil viscosity, and (**c**) gas oil ratio.

Using the absolute permeability of specific lithologies based on available lab measurements, we constructed capillary-pressure curves (Fig. 4) and relative permeability relationships for three rock types: sand, silty sand, and shale. Figure 5 presents the PVT parameters used as inputs for the reservoir simulation.

## Results and discussion

### Facies modelling

**Figure 6:**
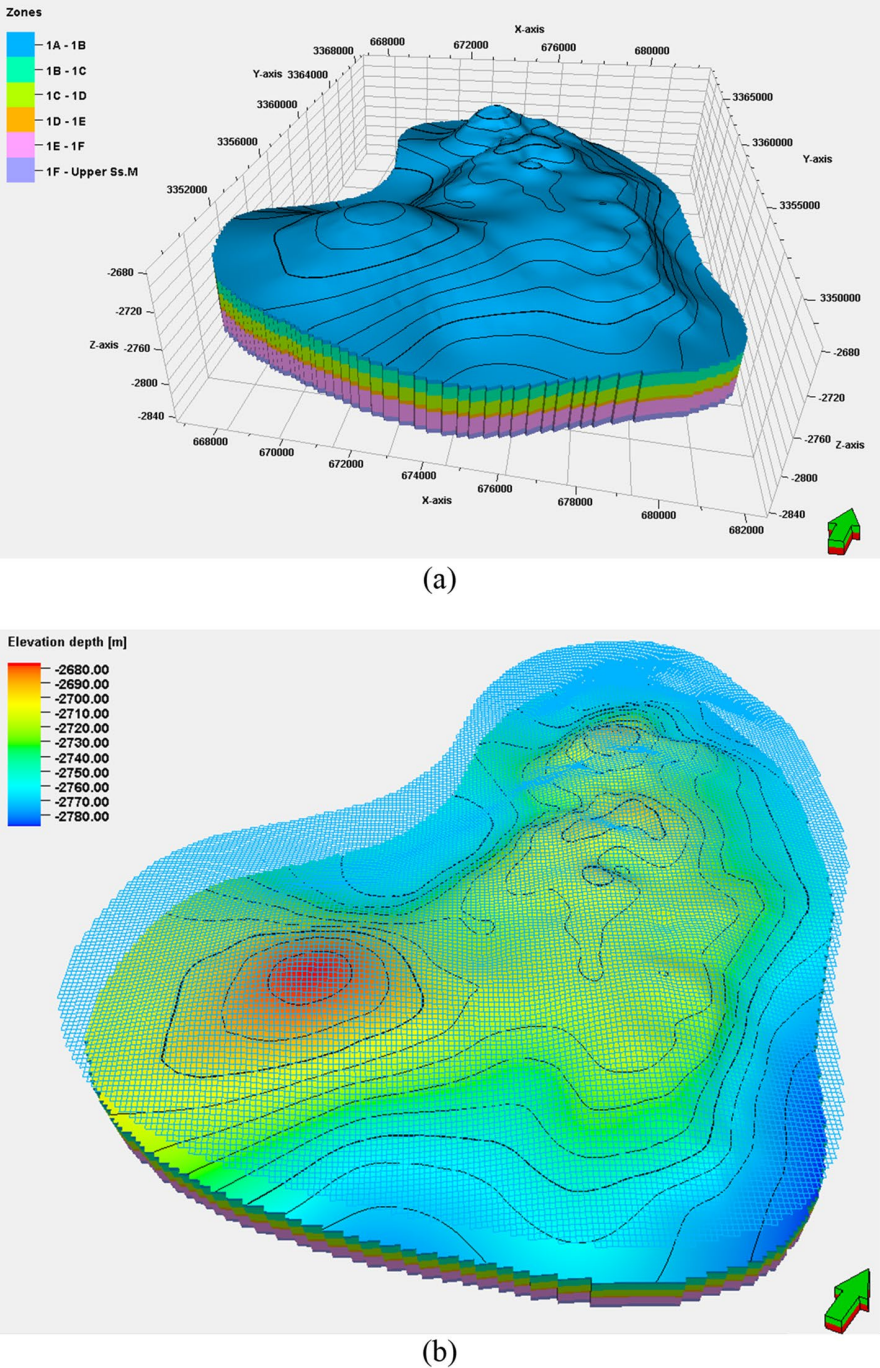
Representations from the grid-based and horizon-based models for the Luhais oil field: (**a**) upper shale 3D structural grid of the component layers and (**b**) contoured structural map of one of the layer surfaces extracted from the 3D grid model.

We analyzed data from 43 wells in the Luhais oil field's upper shale member using multiple statistical methods to predict lithofacies and porosity–permeability distributions. To create the 3D reservoir model, we first established a grid and horizon-based structural framework. We used a 100 m by 100 m grid size for the upper shale member, resulting in 173 × 209 grid units in the I and J directions. The total active grid units amounted to 1,448,202. In the horizon modeling, the main reservoir has six zones and in order to capture the more realistic geological structure, the zones have been subdivided into 40 layers with approximately 1.5 m thickness for each layer. The horizon model and structural gridded surface for the upper shale member in the Luhais oil field are presented in Fig. 6a and b.

**Figure 7:**
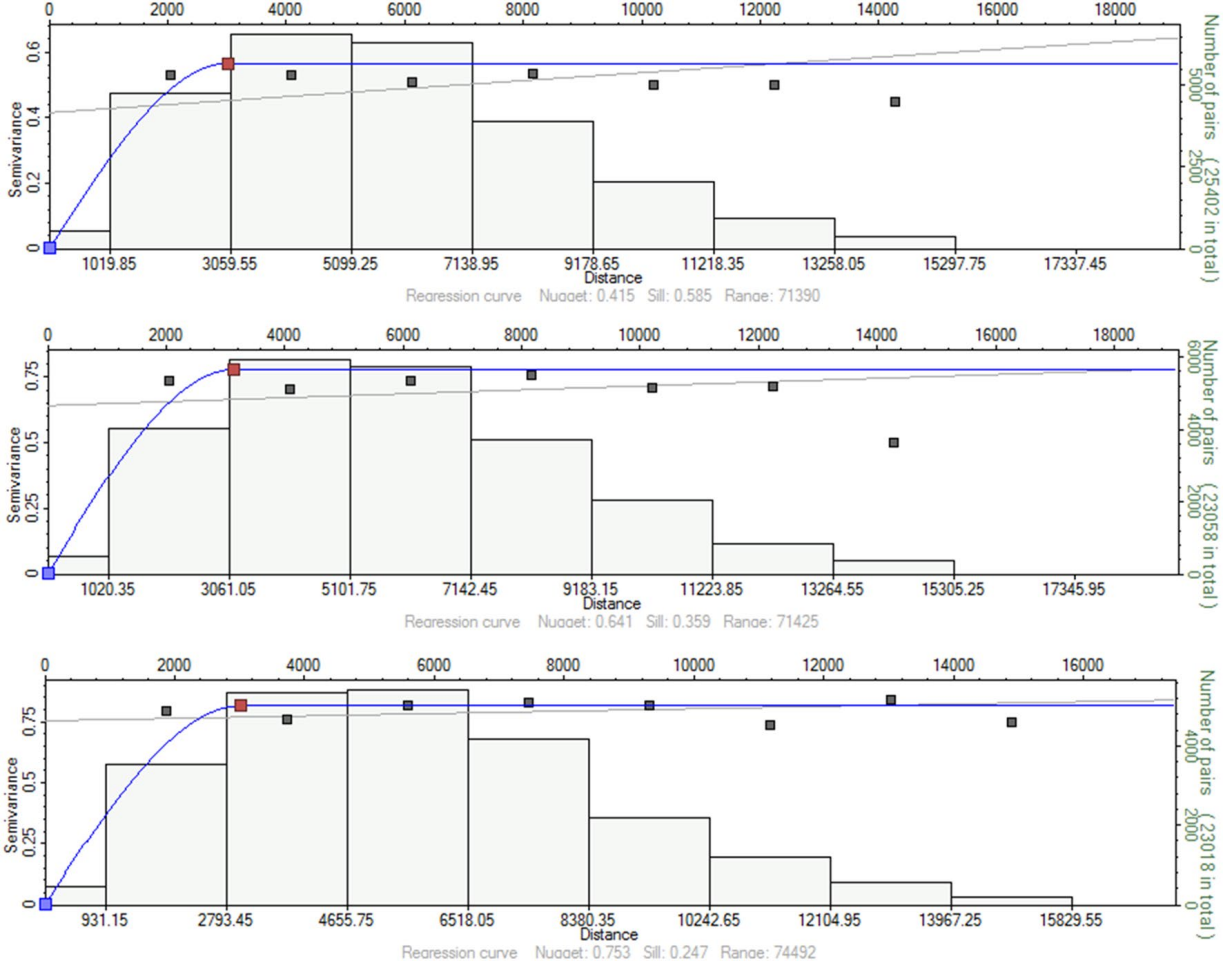
Indicator variogram fitting of the lithofacies: sand (top), silty sand (middle), and shale (bottom) of the Upper Shale Member of the Luhais oil field.

To determine the spatial relationship of each lithotype at an angle of 0° (i.e., horizontal), the SISIM model employed indicator kriging, establishing the spatial association of each lithotype for a specific distance interval (i.e., the lag distance) in this direction. In this context, the total number of lags in all scenarios was 20, with an average range of 3000 m. These indicator variogram settings were selected to incorporate as many available sampled wells as feasible to ensure a dataset large enough to reduce uncertainty in the predicted values for any unsampled grid. In particular, acceptable variogram models were built by modifying an initial empirically specified variogram. Modifications were conducted by assessing the sill (the value at which the model flattens; representing the maximum variance between the observed variables), the nugget (the value causing the variogram to approximately intercept the y-axis; representing the variability at small sample distances including estimation errors), and the range (the distance at which the model flattens, representing the lag length over which the variogram achieves the sill value and the correlation becomes zero above that), which are variogram covariance attributes associated with the spatial distribution. The indicator variograms given the three lithofacies of sand, silty sand, and shale are depicted in Fig. 7. Spherical modeling provides the best fit for all the indicator variograms, for all the lithotypes, in all directions. Table 2 illustrates the indicator variogram parameters for the Lithofacies Modeling given the three lithotypes.

**Table 2:** Indicator variogram parameters for the lithofacies: sand, silty sand, and shale.

Lithofacies	Range			Variogram Parameter			
	Major	Minor	Vertical	Sill	Azimuth	Nugget	Variogram Type
Sand	3011	2816	100	0.560	0	0.0	Spherical
Silty Sand	3130	2422	100	0.778	0	0.0	Spherical
Shale	3009	2606	100	0.814	0	0.0	Spherical

**Figure 8:**
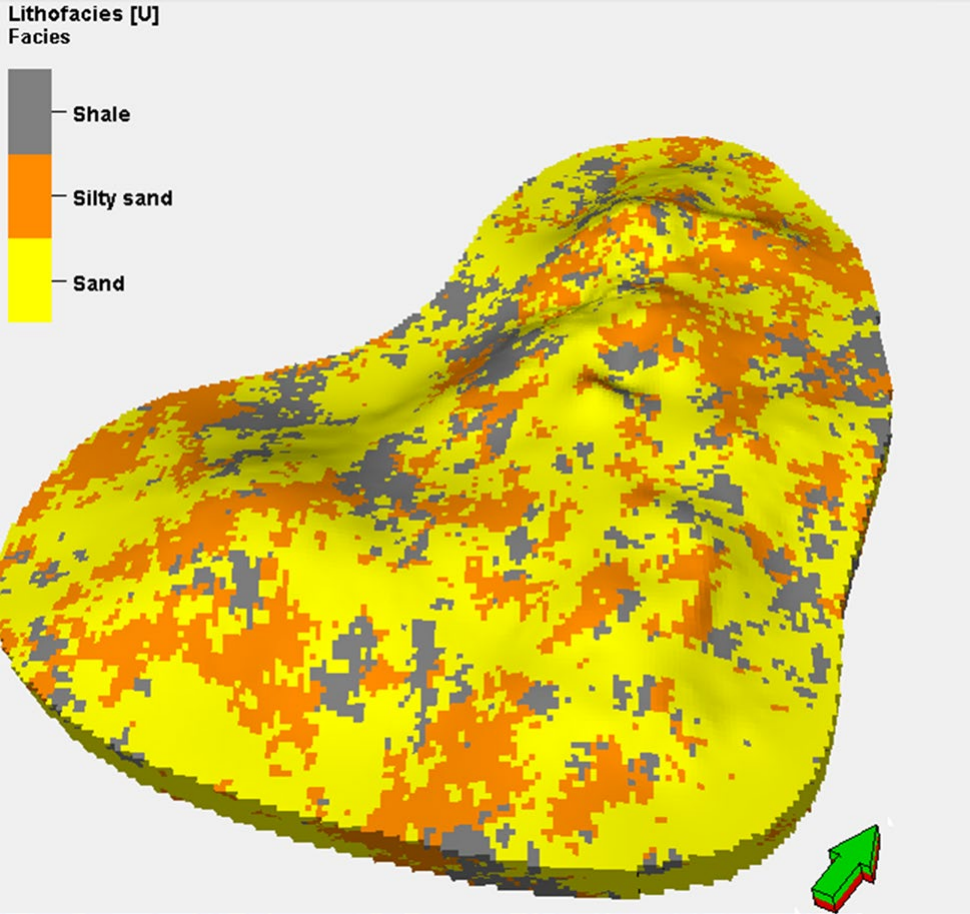
3D geostatistical lithofacies simulation of the upper shale member in the Luhais oil field.

Spherical-influence modeling was used to generate optimum matches for all indicator variograms generated for all lithologies considered in all 3D directions. The sill, nugget, and range from the indicator variogram are required for 3D lithofacies modeling in the SISIM method. The entire dataset, histogram, variogram, and accurate smoothing were evaluated stochastically using SISIM for 3D lithofacies modeling. SISIM was used in preference to the deterministic indicator kriging method (i.e., charting) to improve uncertainty assessment. Figure 8 presents the 3D lithofacies model for the upper shale model.

Before determining the accuracy of the generated 3D lithofacies models for the upper shale member in the Luhais oil field, it was necessary to analyze the reservoir’s sedimentary sequence. Depositional environment analysis revealed that the upper shale component was deposited in predominantly tidal and sand-rich conditions. The SISIM model generated a reasonable representation of the reservoir’s tidal environment. The final SISIM lithofacies model also indicated that shale was widely distributed in the uppermost layers of the reservoir.

### Reservoir characterization

**Figure 9:**
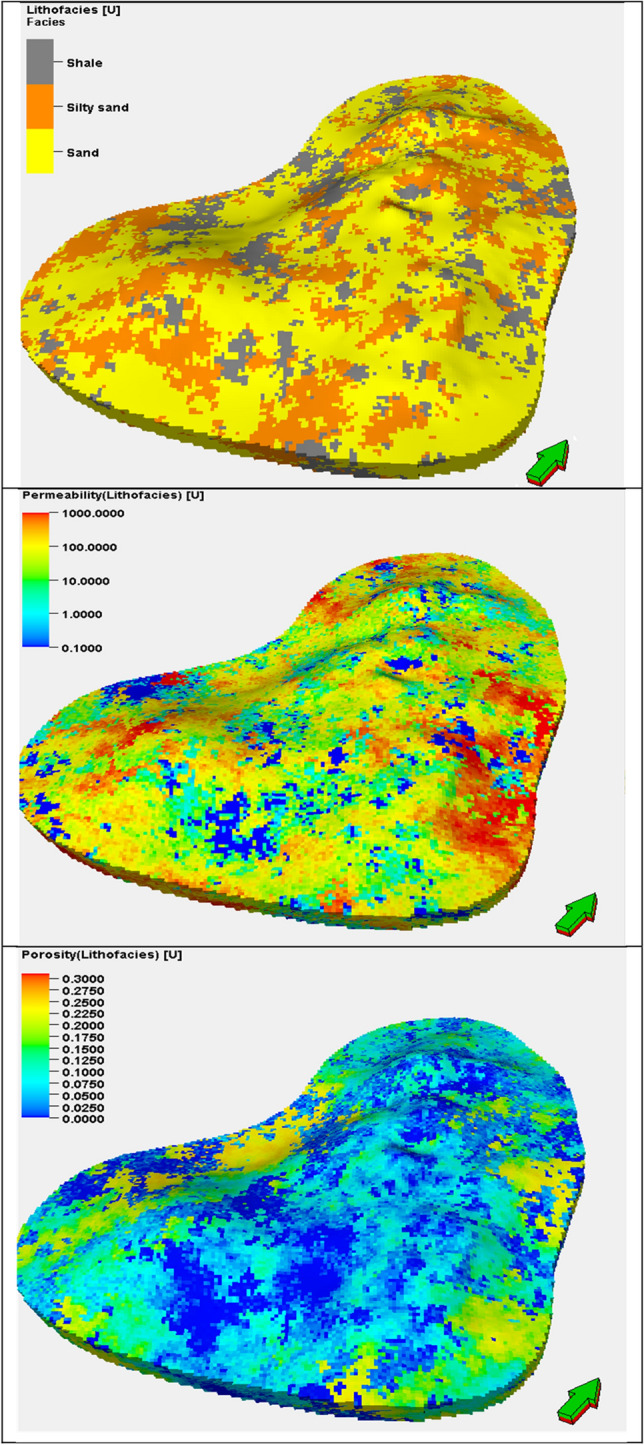
3D geostatistical petrophysical simulation of the upper shale member in the Luhais oil field. The top figure represents the facies based on the petrophysical properties, the middle figure represents the permeability model, and the bottom figure represents the porosity model.

SISIM-based lithofacies modeling for sand, silty sand, and shale was followed by the SGSIM-based modeling of the petrophysical properties (i.e., the porosity and permeability) of each of these rock types. For each rock type, a unique variogram was required based on the differences in the mean and variance of the petrophysical properties because the porosity and permeability of sand are higher than those of shale, with silty sand in between. Once a theoretical variogram model of each rock type was satisfactorily fitted to the experimental variogram values, the optimum variogram values for the sill, range, and nugget and the seed number were then input to the SGSIM for stochastic spatial modeling. Figure 9 displays the 3D geostatistical reservoir characterization output from the SGSIM model for the three rock types and the porosity and permeability.

**Figure 10:**
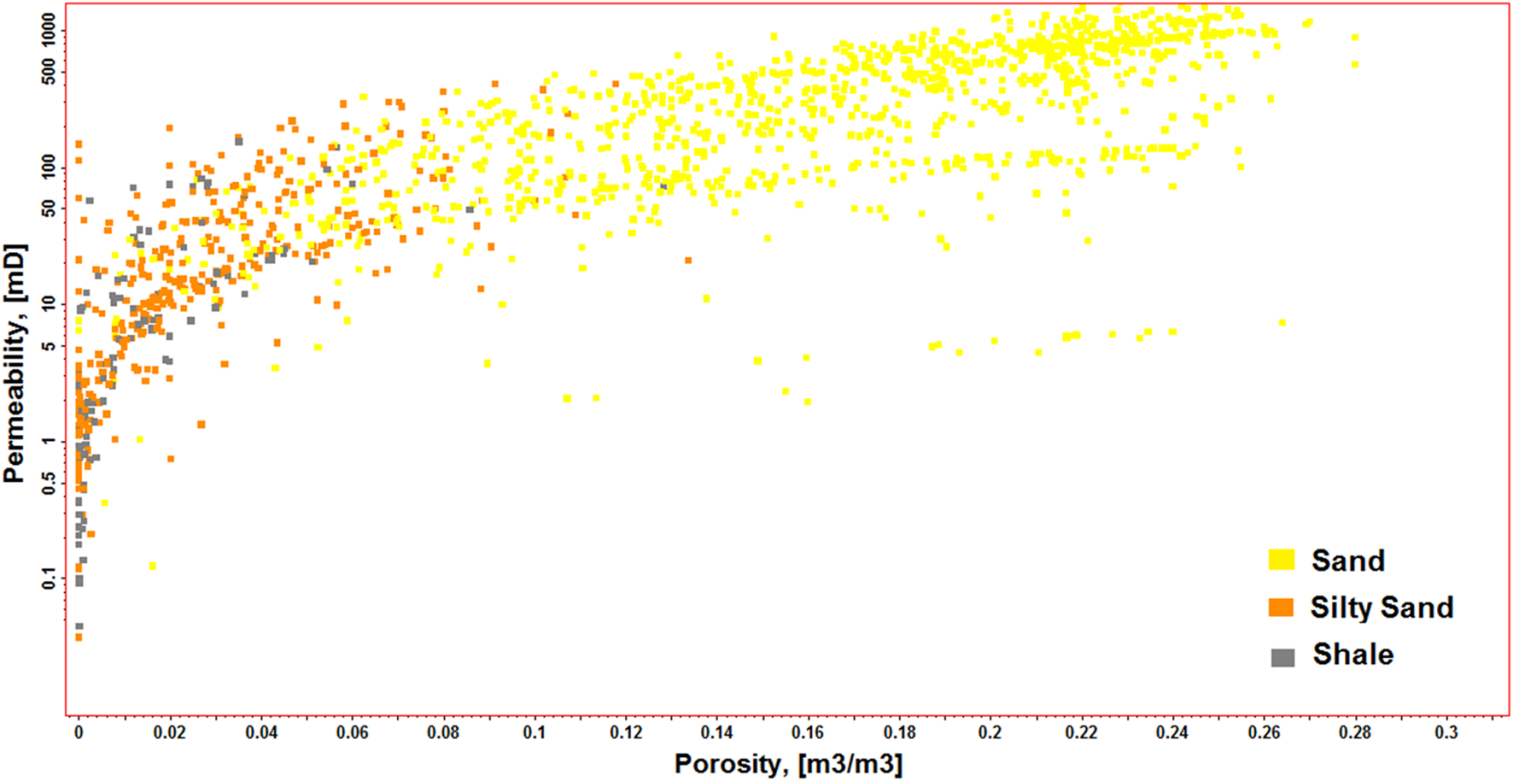
Cross plot between reservoir porosity and permeability for the three lithofacies: sand, silty sand, and shale.

A cross plot demonstrates the relationship between porosity and permeability in connection to lithofacies. This cross plot supports the correlations between high permeability and low permeability zones, which correspond to regions consisting of sand and shale, as depicted in Fig. 10.

Reservoir simulation models consist of a large number of grid cells and time steps, thus they require considerable computing time for each simulation run. For this reason, selecting an appropriate 3D model grid size and scaling factor is essential for the generation of manageable, field-scale reservoir simulation models. This selection decision needs to take into account the money and time available for the analysis and the available computer processing speed. The Luhais oil field has a 41-year production history with 43 producing wells, generating a large set of historical data that requires significant data processing and simulator run times.

**Figure 11:**
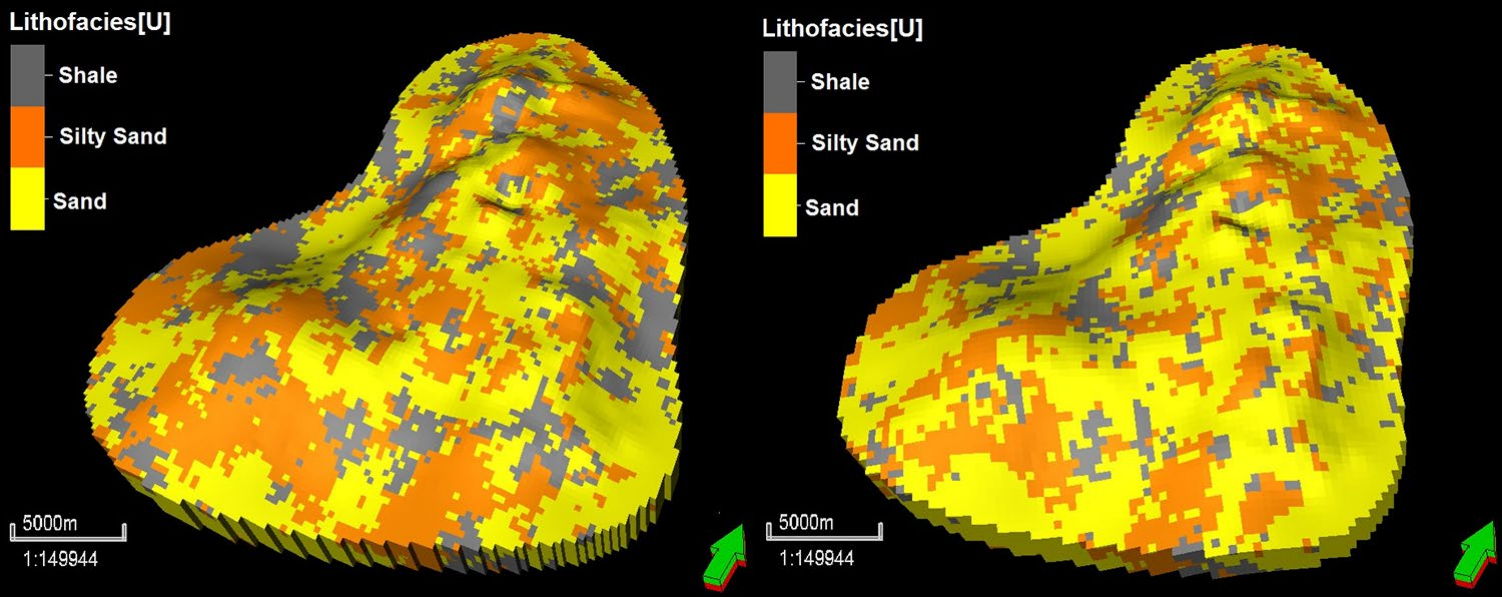
Coarse-scale geostatistical models for the lithofacies at grid dimensions of 150 × 150 m and 200 × 200 m (right).

**Figure 12:**
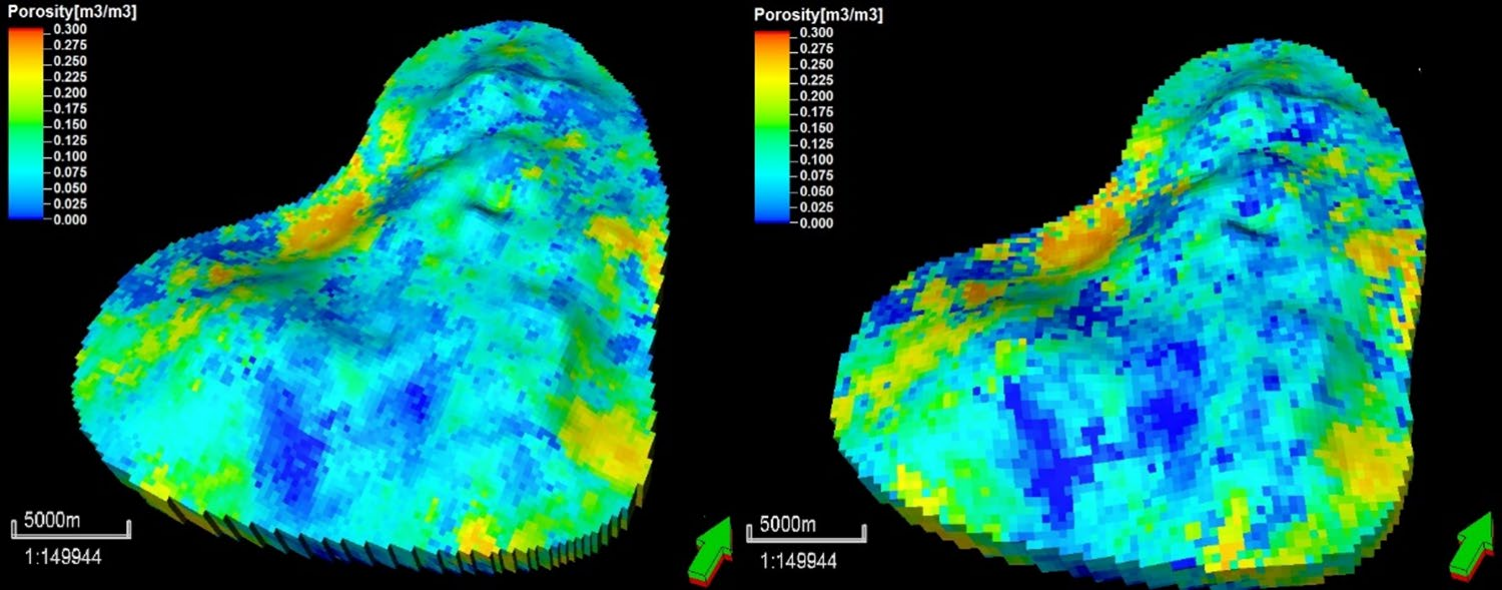
Coarse-scale geostatistical models of porosity according to the lithofacies at grid dimensions of 150 × 150 m (left) and 200 × 200 m (right).

**Figure 13:**
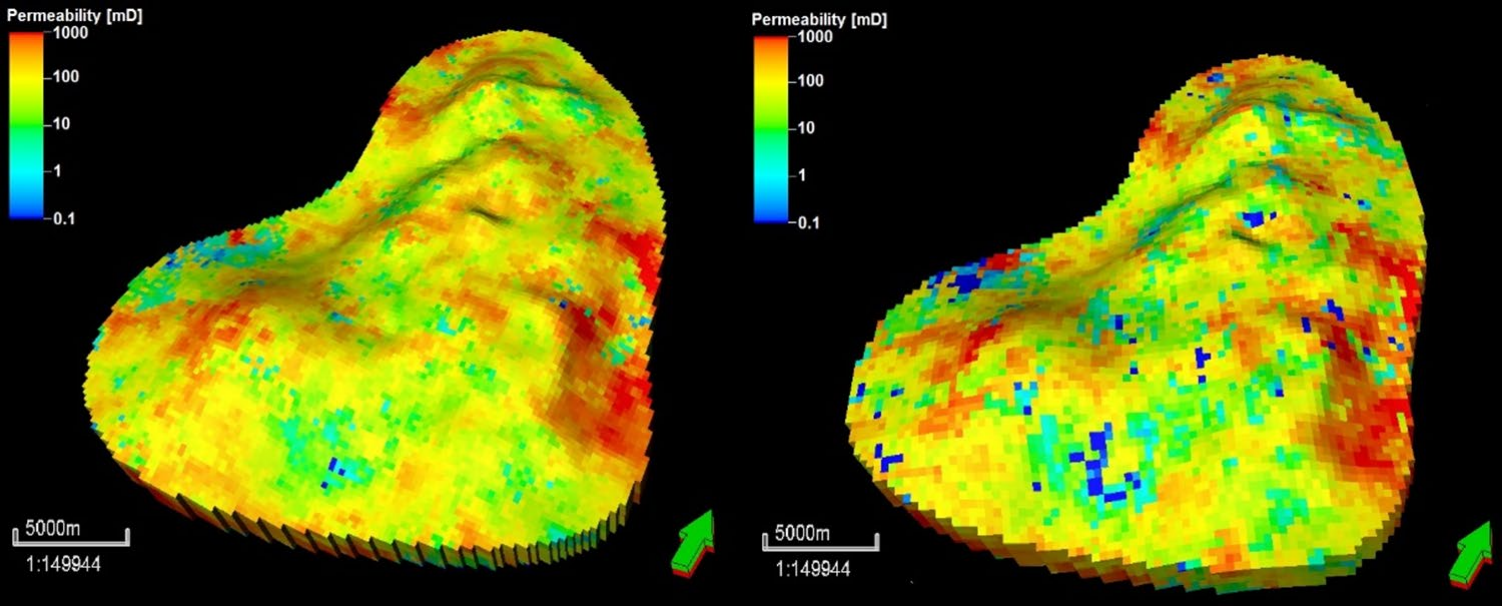
Coarse-scale geostatistical models of permeability according to the lithofacies at grid dimensions of 150 × 150 m (left) and 200 × 200 m (right).

The constructed geostatistical model was upscaled to generate a grid with a lower granularity, with grid dimensions of 200 m × 200 m, in order to reduce the computational time for the full-physics reservoir simulations. This upscaled model contained 86, 105, and 5 grids in the *I*, *J*, and *K* dimensions, respectively, for a total of 45,150 grids. In the vertical dimension, the original 45 layers were reduced to 12. The arithmetic and harmonic means were considered for the porosity and permeability distributions in the sampled wells to assess the impacts of grid upscaling. The quadratic mean or root mean square (RMS) was used for the spatial-property grid upscaling of porosity and permeability. Figure 11 presents the 150 m × 150 m and 200 m × 200 m 3D grid reservoir models for the lithofacies, while Figs. 12 and 13 display the 3D grid reservoir models (150 m × 150 m and 200 m × 200 m) for the porosity and permeability of the lithofacies, respectively.

A comparison of the bulk grid volumes, grid-cell angles, and grid-cell inside-out factors for the 3D models with finer- and coarser-scaled grids was conducted in consideration of other grid attributes. This assessment determined whether the upscaled grid models were of sufficient quality and were able to minimize the simulation error for the pore volume and initial-oil-in-place (IOIP) values. Acceptable percentage discrepancies for these volumes were set at 7%^48^. We obtained the difference in the bulk volume before and after property upscaling of less than 3.5%. In addition, the cell-angle properties of the coarser- and finer-scaled grid models were assessed and the maximum cell-angle deviation from 90° was determined for each model. The acceptable percentage discrepancy for the cell angle was 15°^48^. The cell angle was measured from the inside out to ensure that all reservoir cells had no discrepancies.

**Figure 14:**
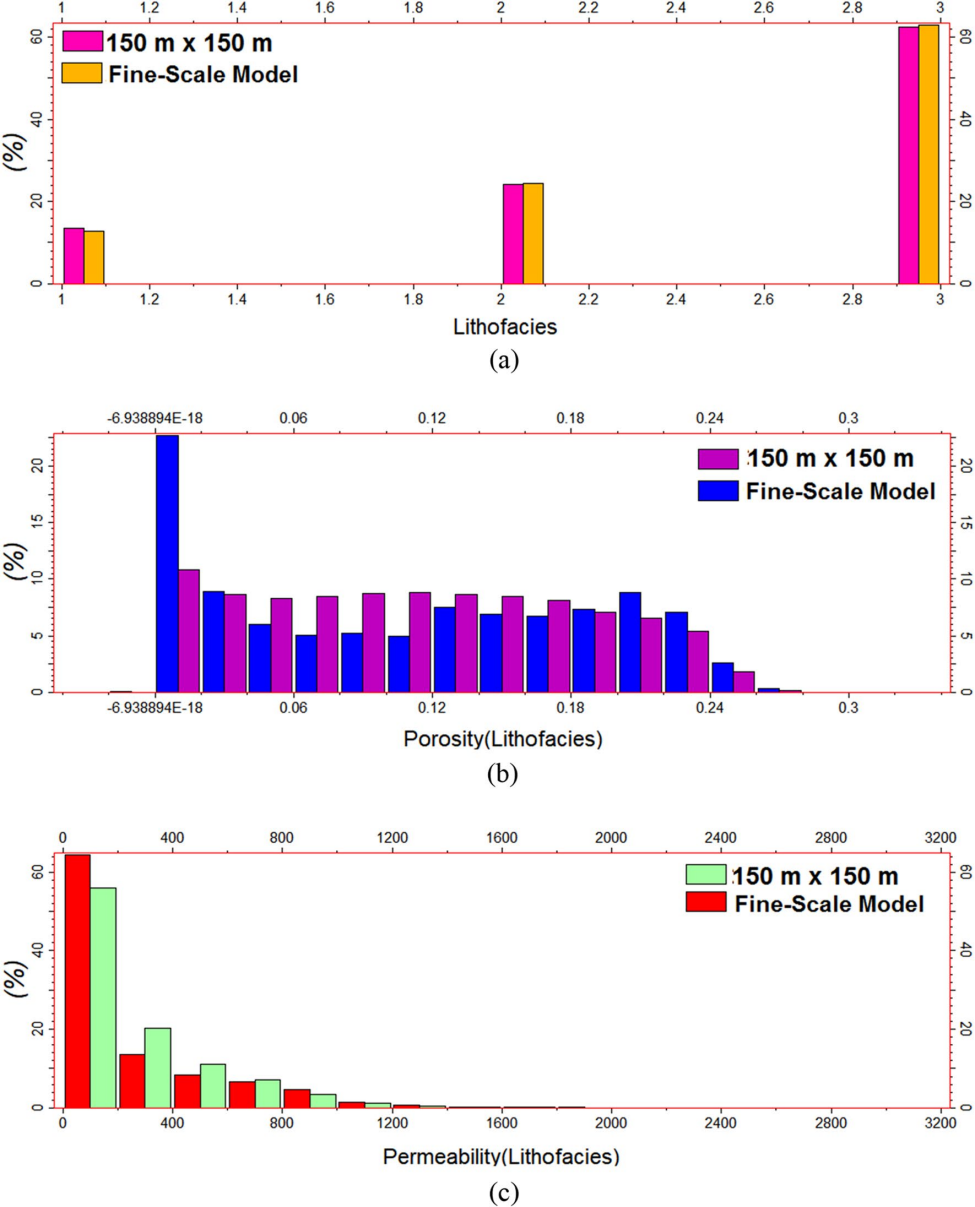
Histogram comparison of the 150 × 150 coarse- and fine-scale models: (**a**) lithofacies distribution and (**b**) porosity and (**c**) permeability distributions based on the lithofacies.

**Figure 15:**
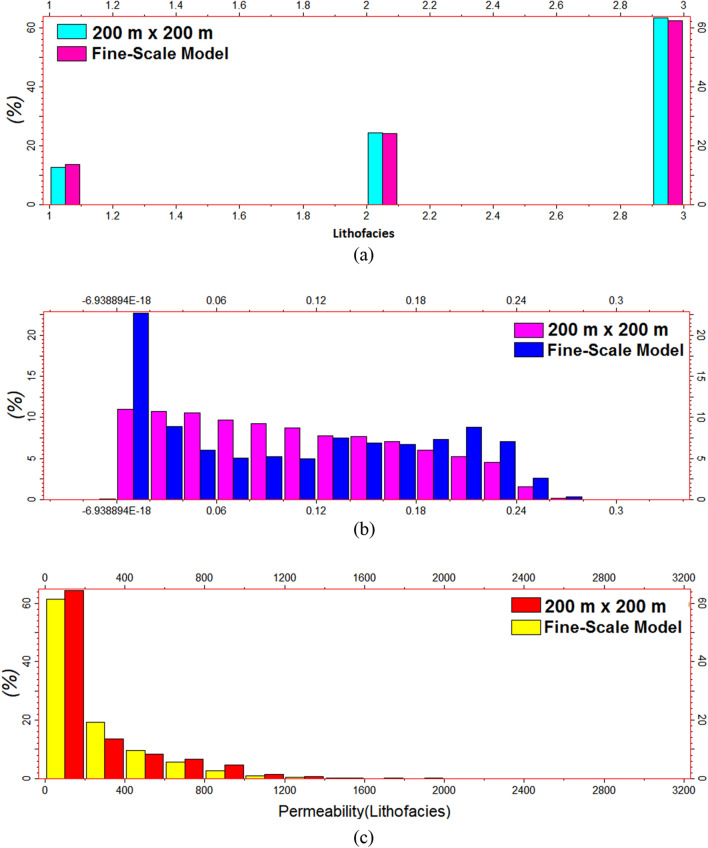
Histogram comparison of the 200 × 200 coarse and fine-scale models: (**a**) lithofacies distribution and (**b**) porosity and (**c**) permeability distributions based on the lithofacies.

A comparison of the histograms for the lithofacies allocations according to the grid size was also employed to assess the reliability of each model. Figures 14 and 15 compare the histograms for the lithofacies, the porosity based on the lithofacies, and the permeability based on the lithofacies for the finer-scale reservoir model with grids of 150 m × 150 m and the coarser-scale reservoir model with grids of 200 m × 200 m, respectively. Both histograms demonstrated a satisfactory performance before and after upscaling. Figure 14b displays a negative value, which corresponds to the lowest porosity data in the fine-scale geostatistical model. The upscaled coarse-scale model (150 m × 150 m) reduces this value to zero. The upscaling approach employs averaging techniques to generate a model at a larger scale, resulting in the elimination of very small negative data points that are near zero. The value of -6.9888E-18 is negligible, resulting in its disappearance in the upscaled reservoir model.

### History-matching

The study aimed to develop a reservoir simulation for the Luhais oil field's upper shale member to assess infill drilling options. Production began in 1976 with well PROD-2, and expanding in 1978 with wells PROD-5 and PROD-7, the reservoir maintained an initial pressure of 4300 psia, which decreased to 4150 psia after 41 years. Ads the pressure remains above the bubble point of 2300 psia the reservoir is classified as an undersaturated reservoir. To date, 20% of its 675 million barrels of oil has been extracted.

To design our simulation, a comprehensive match with historical production data was essential. After matching, the simulation evaluated various drilling scenarios for a 12-year forecast from 2018 to 2030. The simulation's initial step used in-place fluid volumes, reservoir pressure, and fluid-saturation distributions, with specifics provided in Table 3 for the upper shale member as of 31 May 2018.

**Table 3:** Reservoir volume data for the upper shale member in the Luhais oil field. The current volumes and pressures are estimates for 31 May 2018.

Initial reservoir properties	
Average pressure, psia	4300
Average porosity, faction	0.1042
Total bulk volume, res m^3^	5169E+6
Total pore volume, res m^3^	505E+6
Total hydrocarbon pore volume, res m^3^	229E+6
Originally in place at *P*_*i*_ = 4300 psia	
Oil in place, STB	1.037818E+9
Dissolved gas in place, SCF	1.938023E+12
Water in place, STB	3.948989221E+09
Current in place at *P*_*f*_ = 4150 psia	
Oil in place, STB	0.86193E+09
Dissolved gas in place, SCF	1.8285E+12
Water in place, STB	4.1717295E+9
Cumul. water influx, STB	265.8E+6

For accurate historical matches, dynamic data like fluid-production volumes, reservoir pressures, well bottom-hole pressures, water-cut distributions, and more were utilized. The study adjusted lithofacies, permeability, porosity distributions, and aquifer strength for an optimal match. Traditional calibration methods, involving iterative trial-and-error, are lengthy and sometimes fail to address uncertainties in reservoir characteristics. Our approach used a conditional simulation of lithofacies and petrophysical properties, generating multiple reservoir model realizations. The best model had the lowest mismatch error.

**Figure 16:**
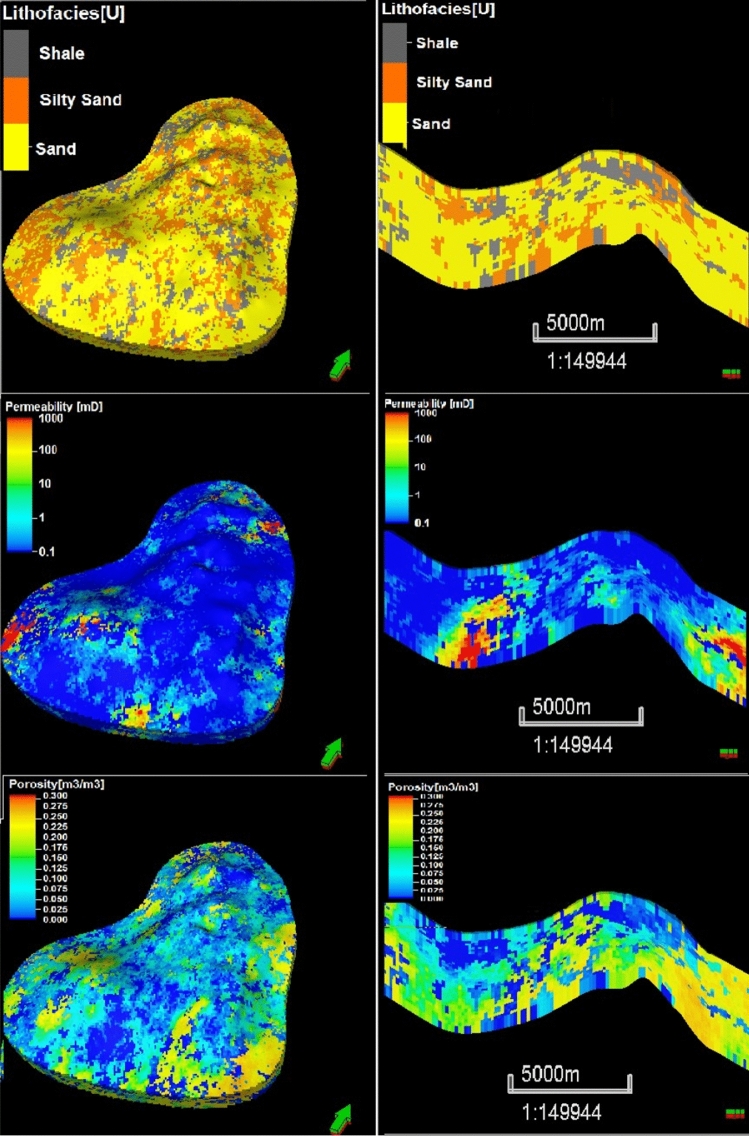
Top and cross-section views of the reservoir provided by the realization with the best simulation history match (i.e., the lowest mismatch error) expressed in terms of the lithofacies, permeability, and porosity spatial distributions.

**Figure 17:**
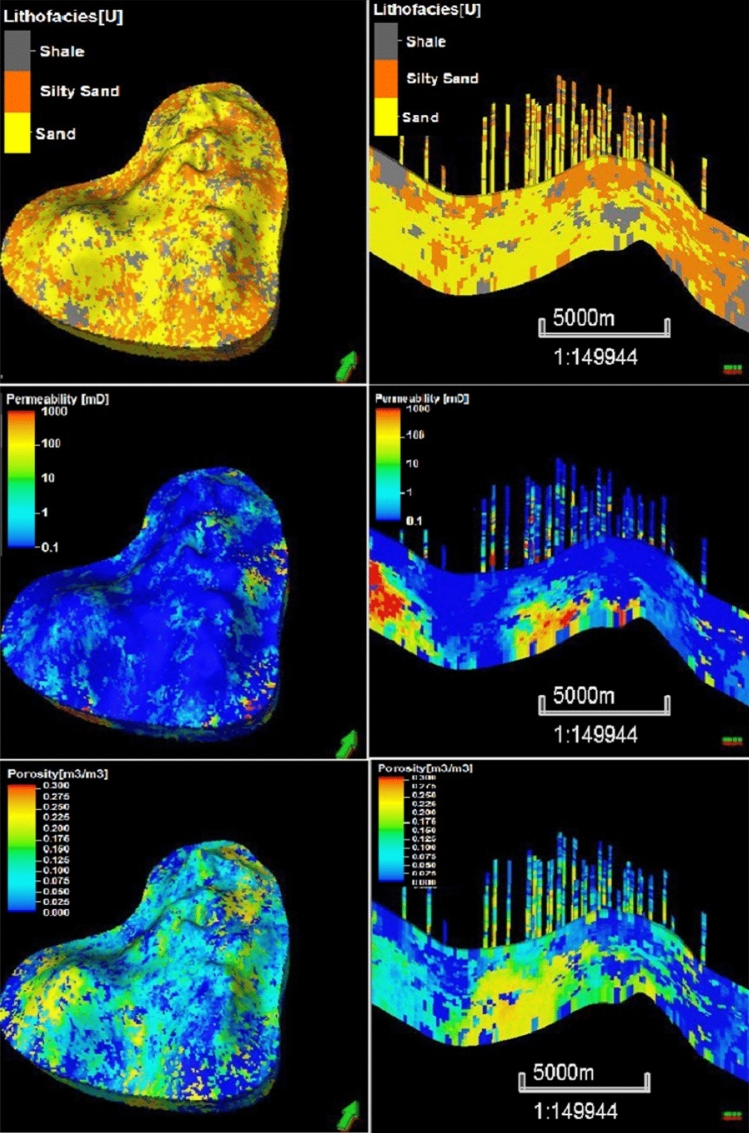
Top and cross-section views of the reservoir provided by the realization with the second-best simulation history match (i.e., the second-lowest mismatch error) expressed in terms of the lithofacies, permeability, and porosity spatial distributions.

Using SISIM, we stochastically crafted 50 cases, each representing different reservoir models for lithofacies. After determining permeability and porosity for each, they were evaluated via a full-physics reservoir simulation. Their quality was gauged by the difference in simulated and actual production profiles. Figures 16 and 17 showcase the best two realizations based on their respective errors and facies, permeability, and porosity distributions.

**Figure 18:**
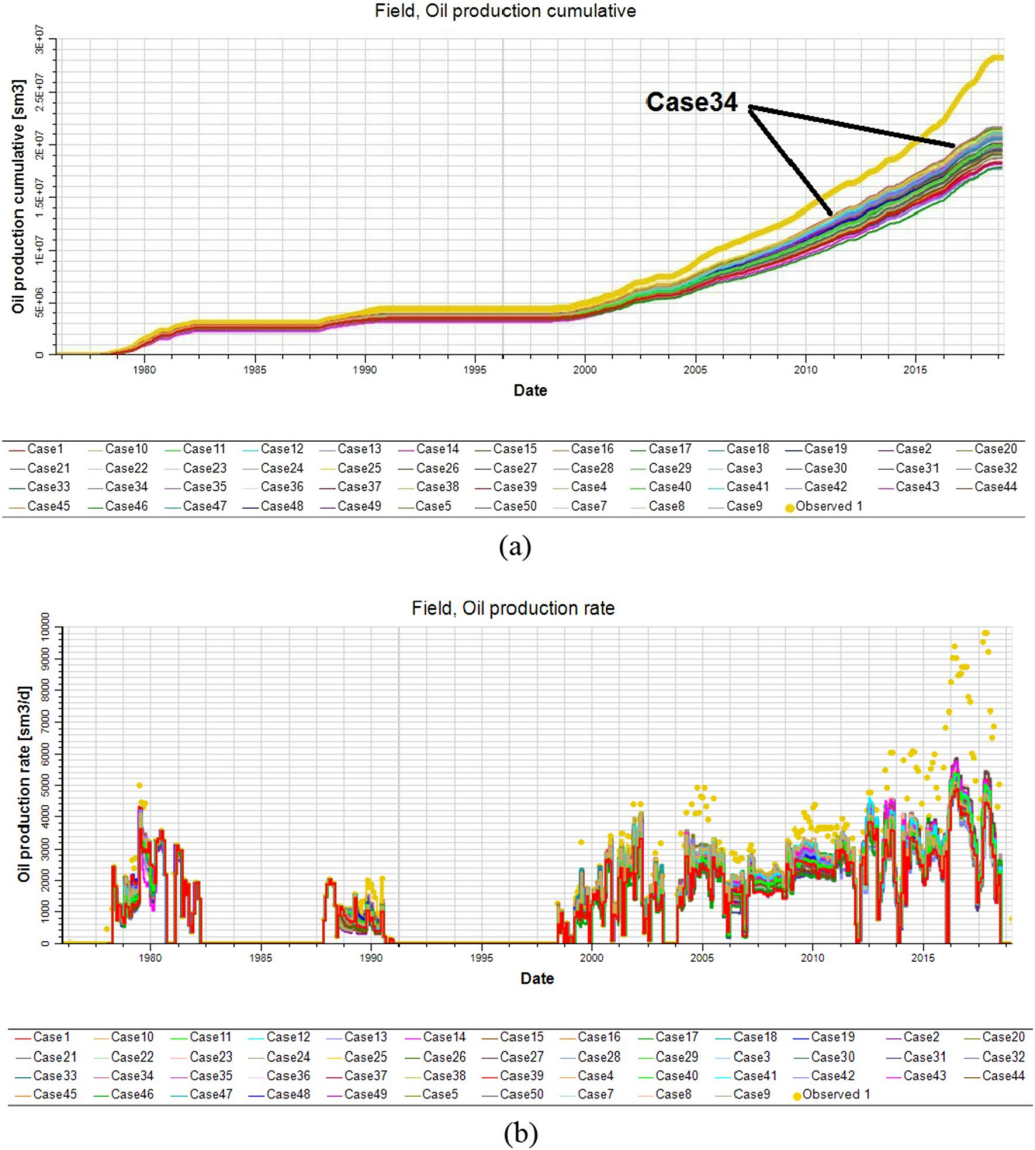
Simulation history match results for the upper shale reservoir for 50 simulation cases expressed in terms of (**a**) the annual cumulative oil production and (**b**) the annual oil production flow rate. The observed data is presented in yellow.

The history matches for each simulation case were assessed in terms of the cumulative oil production and oil flow rates for the upper shale member in the Luhais oil field. In Fig. 18, the 50 cases are displayed together with the historically recorded values. It is clear that the history matches for all cases, particularly those after the year 2000 are poor, substantially underestimating the observed cumulative oil production and oil flow rates for the upper shale reservoir.

**Figure 19:**
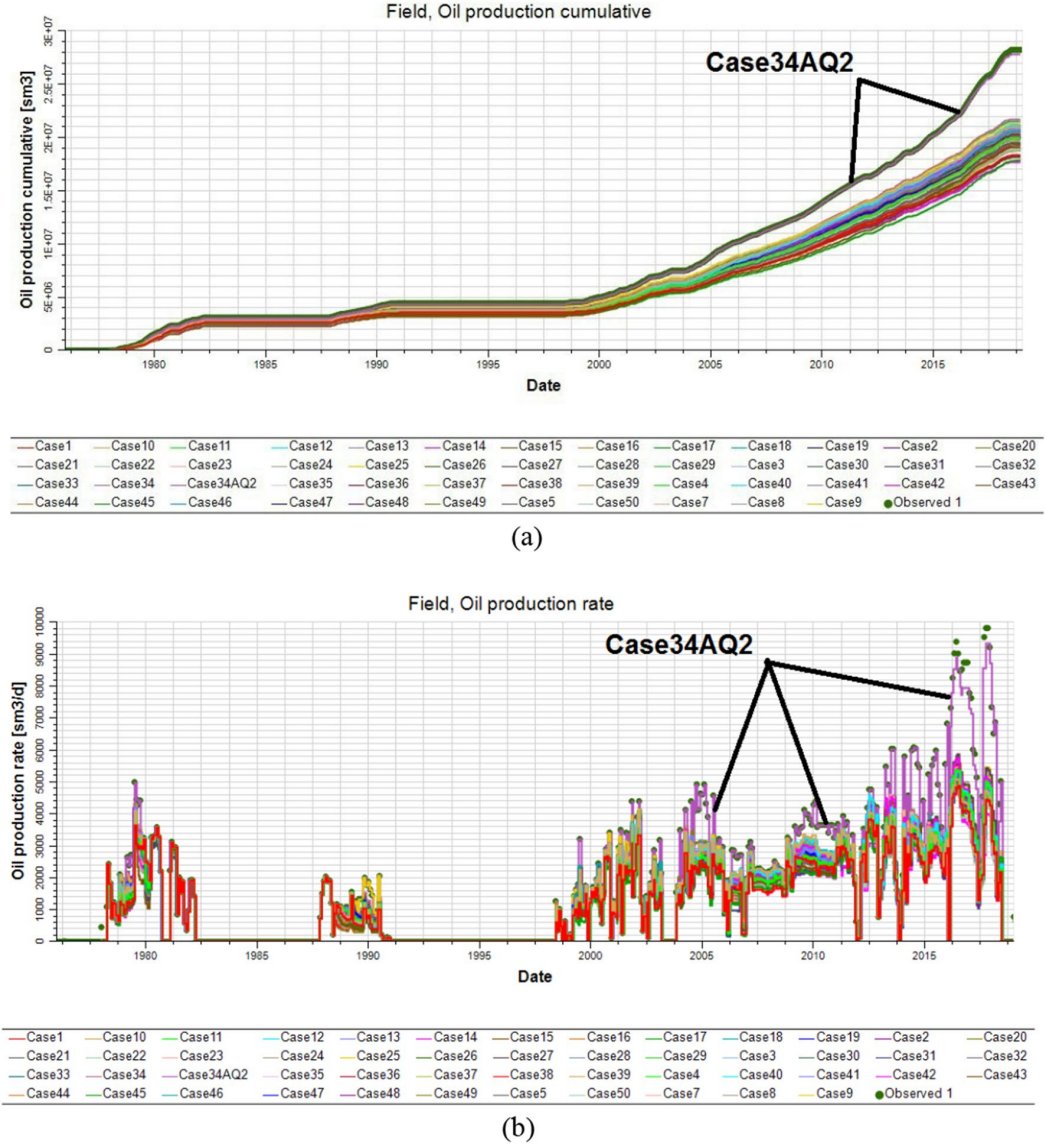
Optimal adjusted solution for the simulator history match results derived from case 34 (purple) for the upper shale reservoir from among 50 simulation cases expressed in terms of (**a**) the annual cumulative oil production and (**b**) the annual oil production flow rate. The observed data is presented in blue.

After running the reservoir model incorporating the 50 realizations of lithofacies, porosity, and permeability, 50 curves of production history were obtained and the base case represents the least mismatch between the observed and predicted reservoir flow responses, as illustrated in Fig. 18a and b. The permeability distribution for the best realization (Case 34 in Fig. 18a) had to be multiplied by 2.5 to close the gap between the observed and predicted cumulative oil production and oil flow rates. This 2.5 multiplier represents the difference between the permeability ranges before and after the well log upscaling. By making this adjustment (Case 34AQ2 in Fig. 19a), an optimal adjusted solution was obtained that accurately matched the observed cumulative oil production and oil flow rates (Fig. 19a and b, respectively).

From Figs. 18 and 19, acceptable history matching was attained by applying geostatistical reservoir modeling and creating multiple realizations for the lithofacies, porosity, and permeability in the reservoir simulation. It would be difficult to achieve satisfactory history-matching results by considering only a single realization because a large degree of uncertainty needs to be accounted for to obtain more realistic results. The best realization should be considered for forward-looking predictions of reservoir performance. In particular, full-field development plans can be adapted to use the best realization from the history-matching models to propose multiple strategies aimed at maximizing the oil recovery factor and reservoir management. Additionally, the best history-matching model can be further utilized for designing the optimal oil production forecast scenario. The well production design could then also be based on history-matching models to monitor the ongoing performance of producer and injector wells in secondary recovery plans. Furthermore, EOR/IOR strategies need to rely on the best history-matching model to reduce the risk and optimize net present value outcomes.

## Summary and conclusions

For reliable generation in oil/gas reservoir models, it is vital to address uncertainties in extrapolating parameters such as lithology and porosity–permeability between sampled areas, mainly from limited data sources such as cores, well logs, and seismic surveys. This uncertainty is significant in complex reservoirs with limited sampling, affecting the accuracy of fluid flow and resource recovery predictions.

3D geostatistical models help to capture spatial geological variations. A practical approach to handle uncertainty is by creating multiple reservoir realizations, each extrapolated from sampled well data. These models can then be adjusted for dynamic reservoir simulations, providing accurate historical matches. The best-matching model aids in future reservoir performance predictions and informs field development decisions, especially for evaluating enhanced oil recovery techniques.

We employed this approach in the case study of the Luhais oil field’s upper shale reservoir in Iraq. Through 50 realizations, we highlighted the importance of addressing uncertainty and improved history-matching models. This method also facilitated optimal field development planning and production, enhancing economic returns and devising recovery strategies. P10, P50, and P90 models offer confidence boundaries for future actions and guide potential carbon storage field activities.

## Data Availability

The data that support the findings of this study are available from Basrah Oil Company but restrictions apply to the availability of these data, which were used under license for the current study, and so are not publicly available. Data are however available from the co-corresponding author Watheq J. Al-Mudhafar (watheq.almudhafar@utexas.edu) upon reasonable request and with permission of Basrah Oil Company.

## Abbreviations

*API*: American Petroleum Institute
Perf (m): Perforation intervals in meter
*BHP* (psia): Bottom hole pressure

## List of symbols

*Pb* (psia): Bubble point pressure
*T* (°F): Reservoir temperature
*GOR* (SCF/STB): Gas oil ratio
*Bo* (bbl/STB): Oil formation volume factor
*I*(*Z*_*k*_; *x*): Random indicator (*I*(*Z*_*k*_; *x*) has an expected value equal to the accumulated likelihood *Pr*{*Z*(*x*) < *Z*_*k*_} of its random indicator)
*Z*(*x*): Random function
*Z*_*k*_: Assigned threshold value
*γ*(*h*): Variogram at *h* lag distance
*N*_*h*_: Number of pairs of measurements
*Q*(*x*): Mismatch error (observed versus simulated) for history-match variable *x*
*w*_*i*_: Mismatch weight factor
*Y*^*obs*^: Distribution of observed values of variable *x* (*n* observations)
*Y*^*cal*^: Distribution of simulated values of variable *x* (*n* observations)
